# Supplementary material for: Microsatellite Dataset for Cultivar Discrimination in Spring Orchid (Cymbidium goeringii)
Source: Genes (Basel). 2023 Aug 11;14(8):1610. doi: 10.3390/genes14081610 (PMC10454716; doi:10.3390/genes14081610)
Supplement: Supplementary file 1 [file genes-14-01610-s001.zip › genes-2536089-supplementary.pdf]

Table S1. Combined genotypes of 12 SSRs observed in *C. goeringii* cultivars

Cultivars	CG[1]	Frequency (%)	Genotype of SSR marker[2]												CMP
			CG415	CG649	CG709	CG722	CG787	CG1023	CG1028	CG1085	CG1210	CG1281	CG1320	CG1400	
Hwanggeumso	CG1	64.7	11-11	15-30.1	17-18	12-20	24-29	13-13	15-17	14-15	29-29	16-16	10-13	13.1-20	2.369×10^{-15}
	CG2	10.7	11-13	21-30.1	18-18	12-20	24-24	13-13	15-15	14-19	15-29	16-16	10-13	13.1-20	7.809×10^{-15}
Gwaneum	CG1	57.5	11-13	21-30.1	18-18	12-20	24-24	13-13	15-15	14-19	15-29	16-16	10-13	13.1-20	7.809×10^{-15}
	CG2	10.0	15-15	14-15	17-17	12-17	24-24	13-13	17-17	15-15	15-17	8-8	7-12	13.1-13.1	1.346×10^{-14}
Boreumdal	CG1	62.0	11-20	15-15	17-25	17-17	24-24	13-13	6-7	14-14	6-6	16-16	10-10	15-20	1.894×10^{-15}
	CG2	6.4	11-11	21-21	17-17	12-12	24-24	20-20	17-17	14-14	17-17	10-10	7-7	20-20	4.542×10^{-15}
Deungdae	CG1	56.2	11-15	16-16	17-17	12-12	24-24	13-13	7-19	14-38	15-29	16-16	12-12	20-21	1.521×10^{-17}
	CG2	8.8	10-11	15-21	22-25	17-17	17-17	13-20	12-12	14-17	6-6	16-16	7-7	19-19	2.693×10^{-19}
Manmool	CG1	74.5	13-13	15-29.1	23-23	12-17	18-18	13-20	15-17	19-19	17-17	9-9	12-12	20-20	4.818×10^{-20}
	CG2	1.6	13-13	15-25.1	22-22	12-12	24-24	20-20	29-29	14-14	15-17	10-10	7-7	19-19	1.408×10^{-16}
Cheonhwangso	CG1A	41.0	11-19	14-15	17-34	12-12	24-24	13-13	17-17	15-15	12-12	16-16	12-12	20-20	1.483×10^{-13}
	CG1B	29.8	11-11	15-29.1	17-34	12-20	24-24	13-25	17-17	16-16	17-17	16-16	7-12	13.1-20	1.970×10^{-14}
	CG2	3.7	11-13	21-30.1	18-18	12-20	24-24	13-13	15-15	14-19	15-29	16-16	10-13	13.1-20	7.809×10^{-15}
Daehongbo	CG1	61.8	13-22	15-15	17-18	20-20	24-24	13-13	17-17	Null	15-15	16-16	7-7	19-19	8.890×10^{-10}
	CG2	6.3	13-13	15-15	17-25	12-13	24-24	13-18	7-7	14-14	12-12	10-16	10-10	13.1-20	4.601×10^{-16}
Cheonsoo	CG1	70.6	11-13	14-15	18-18	17-20	24-24	20-20	17-17	14-14	15-15	10-16	7-12	20-20	7.999×10^{-12}
	CG2	7.0	12-20	17-17	18-22	11-11	23-23	17-17	16-23	14-20	14-27	29-29	12-12	23.1-23.1	4.192×10^{-33}
Cheonjong	CG1	76.0	11-11	15-19	17-17	12-20	24-24	13-13	6-30	14-14	15-15	9-9	10-15	13.1-20	1.742×10^{-14}
	CG2	4.0	11-13	15-19	17-23	20-22	24-24	13-13	17-30	14-14	15-15	9-9	10-10	20-23.1	5.161×10^{-15}
Hongdaewang	CG1	51.4	13-13	15-33.1	17-22	12-20	24-24	19-19	30-30	14-14	6-15	16-16	6-7	19-20	1.152×10^{-14}
	CG2	7.3	11-15	23-34.1	17-22	11-11	24-24	13-13	15-17	22-22	15-17	9-16	7-7	17-19	2.363×10^{-16}
Cheonunso	CG1	57.1	13-13	15-15	18-18	30-30	Null	26-26	17-17	14-14	15-15	16-16	7-7	10.1-13.1	3.505×10^{-17}
	CG2	4.1	13-15	15-15	17-25	12-17	24-24	13-13	17-17	14-14	15-15	16-16	6-7	19-20	6.306×10^{-10}
Hwansaeng	CG1	70.5	13-13	26.1-30.1	18-18	11-11	Null	13-13	6-12	14-15	17-29	16-16	7-9	20-20	8.045×10^{-16}
	CG2	5.3	10-11	15-21	22-25	17-17	17-17	13-20	12-12	14-17	6-6	16-16	7-7	19-19	2.693×10^{-19}
Wonmyoung	CG1	91.4	11-13	15-15	22-22	11-12	24-24	13-20	17-17	15-15	15-15	16-16	12-12	13.1-13.1	4.943×10^{-12}
	CG2	1.1	11-15	25.1-25.1	14-24	20-27	Null	12-12	7-7	9-9	6-8	11-12	12-12	15-19	1.168×10^{-33}
Irwolhwa	CG1	61.5	11-19	15-15	17-34	12-17	22-24	13-13	15-15	14-14	15-33	9-9	7-7	19-19	1.346×10^{-14}
	CG2	11.0	15-22	15-15	17-22	12-12	22-22	13-13	17-30	14-15	15-15	9-17	12-12	20-20	1.151×10^{-13}
Taehongso	CG1	73.6	11-13	15-20	17-17	12-20	22-22	20-20	6-6	14-14	15-28	9-16	12-12	15-15	2.074×10^{-17}
	CG2	3.3	13-22	20-20	17-17	12-12	25-25	13-20	17-25	14-15	15-15	16-16	6-7	20-20	3.576×10^{-16}
Munsubong	CG1	61.9	13-13	25.1-29.1	17-22	12-12	22-24	20-20	15-17	14-19	12-15	16-16	7-12	13.1-18	8.785×10^{-15}
	CG2	7.1	13-22	14-14	16-32	17-17	18-22	13-13	17-29	Null	12-15	10-16	12-12	19-19	2.557×10^{-17}
Agassi	CG1	65.4	13-22	15-15	17-17	12-20	24-24	13-20	15-17	16-16	6-15	10-17	7-12	19-19	1.209×10^{-14}
	CG2	9.0	12-13	15-29.1	12-17	11-12	22-24	13-13	15-17	14-16	17-27	9-16	7-10	19-20	2.862×10^{-14}

Geummaek	CG1	77.9	13-13	15-29.1	17-22	11-12	22-25	13-20	6-12	19-19	12-15	10-16	10-12	19-20	2.614×10^{-15}
	CG2	5.9	13-13	32.1-32.1	24-25	12-20	18-24	13-13	15-17	Null	8-15	16-16	7-12	10.1-21	4.902×10^{-18}
Haeoreum	CG1	80.6	11-13	14-14	25-25	11-11	25-25	20-20	17-17	14-14	17-17	9-9	7-7	13.1-13.1	8.255×10^{-17}
	CG2	6.0	11-22	29.1-33.1	17-22	12-12	17-24	28-28	15-30	14-19	15-15	16-16	10-10	23.1-23.1	8.350×10^{-18}
Okro	CG1	89.4	11-13	15-20	17-17	12-22	24-24	13-13	7-17	14-15	12-15	16-16	6-12	13.1-13.1	2.567×10^{-12}
	CG2	3.0	11-18	15-15	13-18	21-21	16-16	20-20	6-26	15-15	9-15	9-26	12-12	14-23	1.993×10^{-27}
Sagye	CG1	80.0	13-15	15-26.1	18-26	12-12	16-22	13-25	17-30	14-15	6-17	9-16	7-12	15-15	3.772×10^{-18}
	CG2	1.5	13-15	28.1-28.1	13-13	12-12	18-18	20-20	12-19	14-14	15-15	10-10	12-12	19-19	9.950×10^{-24}
Chaeun	CG1	74.6	13-13	29.1-29.1	16-16	12-12	22-24	13-13	15-15	14-14	17-17	9-16	7-13	13.1-20	5.165×10^{-15}
	CG2	1.7	11-13	15-25.1	17-18	11-12	24-24	13-20	17-17	14-14	6-15	9-16	7-12	19-20	5.317×10^{-10}
Cheongeumso	CG1	81.0	13-19	15-29.1	17-25	12-17	24-25	13-13	17-29	14-24	15-15	16-16	7-7	13.1-19	4.141×10^{-14}
	CG2	3.4	11-15	15-24.1	18-34	20-22	24-25	13-20	15-15	15-20	6-29	9-16	12-13	13.1-13.1	8.249×10^{-20}
Seongyeong	CG1	72.4	11-11	15-29.1	13-17	12-20	17-24	13-13	17-17	14-14	15-29	9-16	6-6	20-20	1.282×10^{-13}
	CG2	7.0	13-19	15-15	18-22	20-22	22-22	13-13	7-17	14-14	6-17	16-17	15-15	19-19	1.524×10^{-17}
Wonhongseol	CG1	86.0	13-13	16-34.1	13-17	12-17	24-24	13-20	7-17	14-19	11-17	9-9	7-7	13.1-20	6.960×10^{-16}
	CG2	3.5	11-13	13-14	16-19	14-19	14-16	10-23	6-7	11-11	9-28	11-11	5-5	18-25	1.212×10^{-48}
Donglyeon	CG1	60.7	15-15	15-15	17-25	12-20	22-24	13-13	15-15	14-14	15-15	16-16	12-12	20-20	6.555×10^{-12}
	CG2	5.4	13-15	20-20	22-22	12-12	24-24	13-20	7-15	14-14	15-29	9-16	7-7	19-20	1.466×10^{-13}
Jinna	CG1	78.2	10-13	14-29.1	18-18	11-17	Null	13-13	7-7	14-14	15-15	16-16	7-7	19-19	1.674×10^{-12}
	CG2	3.6	12-22	17-17	13-19	12-22	25-25	20-20	7-7	15-15	6-29	9-10	10-12	20-28	1.067×10^{-24}
Jeonju-Hwangu	CG1	69.8	13-15	15-15	34-34	12-20	Null	13-13	7-24	14-14	12-12	16-16	7-7	15-19	2.329×10^{-14}
	CG2	18.9	13-13	14-15	17-22	22-23	Null	19-19	17-17	14-14	6-6	16-16	7-12	10.1-25	1.551×10^{-17}
Cheonbulso	CG1	76.9	11-19	23-26.1	25-34	20-20	24-24	13-13	30-30	14-14	15-15	16-16	12-12	20-20	5.245×10^{-16}
	CG2	5.8	13-13	25.1-25.1	32-32	12-12	22-22	20-20	17-17	14-14	15-15	16-16	7-7	13.1-13.1	5.595×10^{-16}
Bulkkot	CG1	74.5	10-11(10)	15-15	17-17	12-12	24-24	13-13	17-17	Null	12-13(14)	10-16	12-12	19-19	5.094×10^{-13}
	CG2	4.3	13-13	14-25.1	17-22	12-18	24-24	13-13	7-17	9-10	16-27	16-16	10-12	23.1-23.1	1.583×10^{-20}
Hallasan	CG1A	47.5	11-15	15-15	17-17	17-17	24-24	13-13	12-12	14-14	17-17	10-16	10-10	13.1-13.1	3.031×10^{-14}
	CG1B	30.0	11-11	15-15	17-17	17-17	24-24	13-13	17-17	14-14	15-17	16-16	10-10	13.1-13.1	1.720×10^{-11}
	CG2	7.5	11-11	14-14	17-22	12-13	17-24	13-20	17-17	14-14	6-15	16-16	7-7	19-23.1	6.909×10^{-14}
Cheonsa	CG1	59.0	11-13	15-25.1	17-18	11-12	24-24	13-20	17-17	14-14	6-15	9-16	7-12	19-20	5.317×10^{-10}
	CG2	12.8	12-20	17-17	18-22	11-11	23-23	17-17	16-23	14-20	14-27	25-25	12-12	23.1-23.1	9.337×10^{-35}
Susa	CG1	76.3	13-22	14-20	13-22	12-17	15-22	13-13	17-17	14-15	12-15	10-16	7-7	13.1-19	3.073×10^{-15}
	CG2	7.9	11-13	29.1-29.1	17-17	11-17	24-24	13-25	17-17	15-15	17-17	9-16	7-10	13.1-13.1	2.472×10^{-14}
Hongwon	CG1	66.7	13-13	15-15	19-22	12-20	24-24	19-19	30-30	14-14	15-29	16-16	7-7	19-19	8.215×10^{-15}
	CG2	5.6	11-13	15-15	25-25	11-11	Null	13-13	12-15	14-14	29-29	10-16	7-7	13.1-13.1	2.207×10^{-13}
Geumhaso	CG1	75.0	11-19	14-34.1	18-26	12-17	24-24	13-13	6-17	14-19	15-17	16-16	7-13	13.1-20	2.886×10^{-15}
	CG2	6.3	14-15	15-33.1	26-34	11-11	Null	13-13	17-17	14-14	15-15	10-10	7-13	20-20	5.273×10^{-17}
Honggwiin	CG1	63.3	13-13	15-34.1	22-22	12-20	24-24	13-13	7-12	14-14	17-17	16-16	10-10	13.1-13.1	8.121×10^{-14}
	CG2	16.7	13-22	Null	18-20	20-20	20-20	20-20	7-7	22-22	20-20	9-9	12-12	22-22	8.185×10^{-32}

Hwangmae	CG1	75.9	13-13	15-15	17-17	11-22	24-24	13-20	17-30	13-14	15-15	16-16	7-7	20-23.1	1.223×10^{-11}
Migeum	CG1	65.4	11-19	15-15	22-25	11-11	19-24	13-20	6-6	14-14	17-17	10-10	7-7	19-20	1.482×10^{-16}
Dongcheon	CG1	79.1	13-13	15-15	18-18	20-20	24-24	13-13	12-17	14-14	15-15	16-16	7-7	13.1-13.1	7.457×10^{-16}
Unmuryeong	CG1	66.7	13-13	15-29.1	16-17	11-11	17-24	13-20	12-12	14-14	12-12	16-16	10-10	19-19	1.595×10^{-15}
Sacheonwang	CG1	40.9	15-15	15-15	13-13	12-12	24-24	13-13	6-7	14-14	15-15	16-16	7-13	20-20	4.429×10^{-13}
Songjeong	CG1	75.0	11-12	19-30.1	17-18	12-20	17-18	13-13	17-17	24-25	29-30	16-16	13-13	13.1-20	3.106×10^{-24}
Wangjungwang	CG1	56.3	13-15	15-15	18-25	12-22	24-24	13-29	7-17	15-19	15-17	17-17	10-13	13.1-20	1.167×10^{-17}
Hojeong	CG1	53.8	11-11	15-23	13-22	11-12	24-24	13-13	16-16	14-22	15-15	9-9	12-13	13.1-20	4.258×10^{-17}
Saebyeok	CG1	75.0	11-15	15-32.1	17-18	11-22	22-24	13-13	30-30	14-14	15-15	16-16	12-12	19-20	8.840×10^{-14}
Taegeukseon	CG1	63.6	13-15	15-15	17-25	12-17	24-24	13-13	17-17	14-14	15-15	16-16	6-7	19-20	6.306×10^{-10}
Youngchoonso	CG1	81.8	15-15	14-15	17-17	12-17	24-24	13-13	17-17	15-15	15-17	8-8	7-12	13.1-13.1	1.346×10^{-14}
Geumsusan	CG1	45.5	13-22	14-14	17-17	17-20	25-25	13-13	17-17	15-15	15-17	9-9	7-7	19-19	4.971×10^{-15}
Jinjusu	CG1	100.0	12-13	15-29.1	12-17	11-12	22-24	13-13	15-17	14-16	17-27	9-16	7-10	19-20	2.862×10^{-14}
Sinmun	CG1	90.0	13-15	15-29.1	18-18	17-23	24-24	13-13	6-17	14-14	15-17	16-16	7-7	17-20	1.078×10^{-12}
Sebo	CG1	33.3	13-13	20-35.1	18-34	12-22	22-22	13-13	7-18	15-15	17-28	9-16	6-7	13.1-17	1.087×10^{-20}
Silla	CG1	100.0	11-13	20-20	17-17	12-12	24-24	20-20	17-17	14-14	15-15	16-16	7-12	13.1-18	6.238×10^{-14}
Ilchulhwa	CG1	50.0	13-13	32.1-32.1	24-25	12-20	18-24	13-13	15-17	Null	8-15	16-16	7-12	10.1-21	4.902×10^{-18}
Cheongoksan	CG1	100.0	16-16	14-14	19-20	11-20	12-12	13-20	6-6	8-20	13-13	10-10	7-7	20-20	4.496×10^{-36}
Hongun	CG1	100.0	13-13	21-21	18-18	20-20	22-24	25-25	17-17	15-15	15-15	16-16	7-13	20-20	4.016×10^{-17}
Jangdan	CG1	66.6	11-15	23-34.1	17-22	11-11	24-24	13-13	15-17	22-22	15-17	9-16	7-7	17-19	2.363×10^{-16}
Miryeon	CG1	66.7	15-22	14-15	17-22	12-12	22-22	13-13	17-30	14-15	15-15	9-17	12-12	20-20	4.636×10^{-14}
Geumgangbo	CG1	100.0	13-15	15-29.1	18-18	17-23	24-24	13-13	6-17	14-14	15-17	16-16	7-7	17-20	1.078×10^{-12}
Hojin	CG1	100.0	13-13	25.1-25.1	16-16	11-11	25-25	Null	17-17	19-19	12-15	10-16	12-12	19-19	4.440×10^{-18}
Hyangsu	CG1	100.0	13-22	14-14	16-32	17-17	18-22	13-13	17-29	Null	12-15	10-16	12-12	19-19	2.557×10^{-17}
Namhaeso	CG1	50.0	11-11	15-21	13-17	12-20	22-24	13-13	17-17	15-15	15-17	10-15	7-12	17-19	2.578×10^{-15}
Juhongseong	CG1	100.0	13-13	14-25.1	17-22	12-18	24-24	13-13	7-17	Null	16-27	16-16	10-12	23.1-23.1	1.072×10^{-16}
Chanbo	CG1	72.5	12-20	17-17	18-22	11-11	23-23	17-17	16-23	14-20	14-27	29-29	12-12	23.1-23.1	4.192×10^{-33}
Jilbugeum	CG1	92.9	18-18	17-17	18-18	12-12	20-25	21-28	7-23	15-15	15-15	9-9	12-12	23.1-23.1	3.138×10^{-25}
Changseongjihwa	CG1	92.3	20-20	Null	28-28	11-11	18-18	17-17	6-6	9-14	9-27	9-9	7-12	14-23	9.962×10^{-29}
Hobakjeon	CG1	63.6	18-22	17-17	22-22	16-17	17-25	20-28	17-17	20-20	13-15	9-9	7-8	14-23.1	9.868×10^{-28}
Sumunsan	CG1	87.5	19-19	Null	18-19	16-17	25-25	20-20	6-19	15-22	9-9	9-9	12-12	14-23.1	2.083×10^{-25}
Hwanguhajeong	CG1	60.0	11-15	25.1-25.1	14-24	20-27	Null	12-12	7-7	9-9	6-8	11-12	12-12	15-19	1.168×10^{-33}

[1] CG2 profiles were provided for cultivars with a sample number of 30 or more.

[2] Null: failed loci for genotyping.

Abbreviations: CG: combined genotype, CMP: combined matching probability, SSR: simple sequence repeats.
